# Stage-Specific Mortality and Developmental Effects of Synthetic Insecticides, Entomopathogenic Fungi, and Physical Barrier Agents on *Chrysoperla carnea* (Neuroptera: Chrysopidae): Implications for Biological Control in Avocado Orchards

**DOI:** 10.3390/insects17090972

**Published:** 2026-09-21

**Authors:** Michelle Noboa, Arturo Huerta-de la Peña, Cecilia Sue Montiel-Jiménez, Ana Barreiro, Jorge Merino, William Viera-Arroyo, Wilson Vásquez-Castillo

**Affiliations:** 1 Programa de Agricultura y Medioambiente, Universidad Santiago de Compostela, 27001 Lugo, Spain; michelle.noboa@iniap.gob.ec (M.N.); ana.barreiro.bujan@usc.es (A.B.); 2 Programa de Fruticultura, Instituto Nacional de Investigaciones Agropecuarias, Quito 170518, Ecuador; jorge.merino@iniap.gob.ec (J.M.); william.viera@iniap.gob.ec (W.V.-A.); 3 Laboratorio de Control Biológico, Colegio de Postgraduados, Puebla 72760, Mexico; arturohp@colpos.mx (A.H.-d.l.P.); sue.cecilia.mo@gmail.com (C.S.M.-J.); 4 Escuela Superior Politécnica de Chimborazo, Sede Orellana, El Coca 220001, Ecuador; 5 Grupo de Investigación en Alimentos y Agroindustria GIA^2^, Universidad de las Américas, Quito 170513, Ecuador

**Keywords:** lacewing, natural enemies, biorational insecticides, entomopathogens, paraffinic oil

## Abstract

Pesticide contamination of agroecosystems poses significant risks to non-target arthropods, including natural enemies that provide essential pest regulation services. Assessing the ecotoxicological impact of reduced-risk pesticides on beneficial predators is therefore critical for both environmental safety and sustainable agriculture. This study evaluated the lethal and sublethal ecotoxicological effects of six pesticide formulations—abamectin, deltamethrin, paraffinic oil, potassium soap, and the entomopathogens *Beauveria bassiana* and *Metarhizium anisopliae*—on second-instar larvae, third-instar larvae, pupae, and adults of the lacewing *Chrysoperla carnea* under laboratory conditions using topical exposure. Corrected mortality was calculated using Abbott’s formula. Developmental duration and adult longevity were assessed to detect sublethal effects. According to International Organisation for Biological Control classification standards, L2 larvae were the most susceptible stage, where paraffinic oil was moderately harmful, abamectin and *M. anisopliae* were classified as slightly harmful, and deltamethrin was harmless. In pupae, only deltamethrin was slightly harmful, while the remaining treatments were harmless. In adults, paraffinic oil was moderately harmful and abamectin was slightly harmful, whereas potassium soap and both entomopathogens were harmless. Sublethal effects included a significant prolongation of the L2 developmental stage across all treatments and reduced adult longevity following larval exposure to deltamethrin. Overall, L3 larvae and pupae were the most tolerant stages. These results identify potassium soap and the entomopathogens as the most compatible options with *C. carnea*, supporting their inclusion in IPM programmes for avocado orchards, while highlighting the environmental risk posed by paraffinic oil and abamectin to early larval stages and adults of this ecologically important predator.

## Simple Summary

This study assessed lethal and sublethal effects of six reduced-risk pesticides—abamectin, deltamethrin, paraffinic oil, potassium soap, and the entomopathogens *Beauveria bassiana* and *Metarhizium anisopliae*—on the lacewing *Chrysoperla carnea*, a key natural enemy in avocado orchards. Using topical exposure across four developmental stages (L2 larvae, L3 larvae, pupae, adults), researchers found L2 larvae to be the most susceptible stage where paraffinic oil was moderately harmful, and abamectin and *M. anisopliae* were slightly harmful, while deltamethrin was harmful to pupae and adults. Sublethal effects included delayed L2 development and reduced adult longevity. Potassium soap and entomopathogens proved most compatible with *C. carnea*, supporting their use in integrated pest management for avocado.

## 1. Introduction

The widespread and recurrent application of pesticides in agricultural systems represents a major source of environmental contamination that threatens non-target arthropods, including beneficial predators that regulate pest populations naturally [1]. Ecotoxicological assessment of pesticide effects on these organisms is essential to characterise the environmental risks beyond those for target pest species and to support regulatory decisions on pesticide registration and use. *Chrysoperla carnea* (Stephens) (Neuroptera: Chrysopidae) is a generalist predator of soft-bodied insect pests, including aphids, thrips, whiteflies, and lepidopteran eggs [2,3,4] and is widely used as a sentinel non-target species in pesticide risk assessments. Its wide distribution, high predation rate, and ease of laboratory rearing make this insect a standard model for evaluating the ecotoxicological impacts of pesticides on beneficial arthropods in diverse agricultural crops [5,6].

Numerous studies have shown that indiscriminate use of conventional pesticides can seriously compromise the effectiveness of *C. carnea* as a natural enemy, not only through lethal effects but also via sublethal impacts on fecundity, longevity, and behaviour [7,8,9]. Synthetic insecticides such as pyrethroids, carbamates, and organophosphates can cause high mortality rates in larvae and adults of this species [10,11,12]. For example, fenpropathrin and methomyl caused 90–95% mortality in first-instar larvae and more than 50% mortality in adults [11], while abamectin and deltamethrin have also shown high toxicity against larvae and adults of *C. carnea*, with significant effects on mortality, developmental duration, and morphological deformities, especially in larval stages [13]. Even herbicides such as glyphosate have shown adverse effects by altering larval development and morphology in this species [14].

This dependency of risk on both compound toxicity and life-stage susceptibility is particularly relevant in perennial cropping systems subject to sustained and recurrent pest pressure, such as avocado (*Persea americana* Mill.) production. Avocado is one of Ecuador’s fastest-expanding export crops, and its cultivated area has grown steadily in response to increasing international demand, intensifying the frequency of phytosanitary interventions in commercial orchards. In this system, pest pressure from *Stenoma catenifer* Walsingham (Lepidoptera: Depressariidae), *Monalonion velezangeli* Carvalho and Rosas (Hemiptera: Miridae), and *Oligonychus perseae* Tuttle, Baker and Abbatiello (Trombidiformes: Tetranychidae) [15] drives recurrent applications of both synthetic insecticides, such as abamectin and deltamethrin, and biorational alternatives, resulting in repeated environmental exposure of non-target predators such as *C. carnea* to a chemically diverse set of phytosanitary products [16]. Characterising lethal and sublethal toxicity by life stage allows for the identification of critical exposure windows and informs environmental risk assessment frameworks for arthropod non-target organisms [17]. The introduction of *C. carnea* within an IPM approach in avocado therefore requires an accurate understanding of its tolerance to different insecticide groups and the identification of the most susceptible developmental stages, since sensitivity has been shown to vary substantially across life stages, with early-instar larvae generally being the most vulnerable [11,18].

Beyond synthetic insecticides, two additional categories of reduced-risk products are being increasingly incorporated into avocado IPM programmes. Entomopathogenic fungi such as *Beauveria bassiana* (Balsamo) Vuillemin and *Metarhizium anisopliae* (Metschnikoff) Sorokin under residual contact have shown higher selectivity toward *C. carnea*, with larval mortality rates generally below 30%, especially in formulations applied through contact or as dried residues [19]; in those studies, cumulative mortality at 120 h post-exposure was moderate and no significant changes were observed in stage duration or adult emergence, supporting their combined use in IPM [17,19]. Nevertheless, some studies have documented that even these products may affect *C. carnea* depending on the formulation, dose, and treated stage [13,20]. Physical barrier products such as paraffinic oils and potassium soaps act by blocking spiracles, modifying surface tension on pest eggs, or inhibiting oviposition [21,22]; their use in IPM strategies has increased in response to regulatory restrictions on highly toxic pesticides, and they have generally been considered harmless for generalist predators such as *C. carnea* according to IOBC toxicity thresholds [23]. Given that avocado IPM programmes may combine all three of these product categories within a single spray schedule, evaluating them under a common, comparable framework is essential to anticipate their cumulative risk to resident natural enemy populations.

Despite this growing body of literature, three critical gaps remain unaddressed. First, most available studies evaluate a single developmental stage, typically adults or first-instar larvae, precluding stage-specific risk comparisons within the same experimental framework. Second, no study to date has simultaneously assessed lethal and sublethal ecotoxicological effects of synthetic insecticides, entomopathogenic fungi, and physical barrier products across four consecutive developmental stages of *C. carnea* under a standardised IOBC topical bioassay. Third, the ecotoxicological characterisation of these three product categories in the specific context of avocado pest management remains largely absent from the literature, despite the increasing adoption of reduced-risk pesticides in this crop and the ecological relevance of chrysopid predators as biological control agents in Neotropical agroecosystems.

To address these gaps, this study evaluated the lethal and sublethal ecotoxicological effects of two synthetic insecticides (abamectin and deltamethrin), two entomopathogenic fungi (*B. bassiana* and *M. anisopliae*), and two physical barrier products (paraffinic oil and potassium soap) on second-instar larvae, third-instar larvae, prepupae, and adults of *C. carnea* through topical application. Based on previous evidence of stage-dependent tolerance in chrysopids, we hypothesised that early larval instars would show greater susceptibility to all product categories than later developmental stages, and that entomopathogenic fungi and physical barrier products would exhibit comparatively higher selectivity than synthetic insecticides. This work provides an integrated ecotoxicological profile to inform environmental risk assessment and pesticide compatibility decisions within IPM programmes for avocado.

## 2. Materials and Methods

### 2.1. Experimental Conditions and the Rearing of Chrysoperla carnea

All bioassays were conducted in a rearing chamber under controlled conditions of 24 ± 2 °C, 45 ± 4% relative humidity and a 12:12 h (light:dark) photoperiod at the Biological Control of Pests Laboratory of Colegio de Postgraduados, Campus Puebla, Mexico. *Chrysoperla carnea* sensu lato eggs were acquired from the Centro de Reproducción de Organismos Benéficos in Saltillo, Coahuila, Mexico; these insects had not been exposed to pesticides for several preceding generations. Eggs were held in plastic containers (17 cm length × 35.6 cm width × 28 cm height) with paper towel as substrate. Larvae were fed ad libitum with *Sitotroga cerealella* (Olivier) (Lepidoptera: Gelechiidae) eggs obtained from the same centre. Containers were sealed with airtight lids fitted with a filter paper ventilation port.

### 2.2. Bioassay Design

Bioassays followed the guidelines of the IOBC proposed by [24,25,26]. Six low-environmental-impact insecticides plus a distilled water control were evaluated (Table 1). All treatments were prepared at the maximum dose recommended by the manufacturer under field conditions, equivalent to an application volume of 200 L/ha. A surfactant (Inex-A^®^, Cosmocel, Nuevo León, Mexico) was added at a concentration of 1 mL/L to each solution to reduce surface tension and ensure adequate coverage. Two entomopathogen-based products (*B. bassiana* and *M. anisopliae*), two physical barrier products (potassium soap and paraffinic oil) and two synthetic chemical products (abamectin and deltamethrin) were selected; the latter two are considered to have moderate environmental impacts [27].

**Figure 1:**
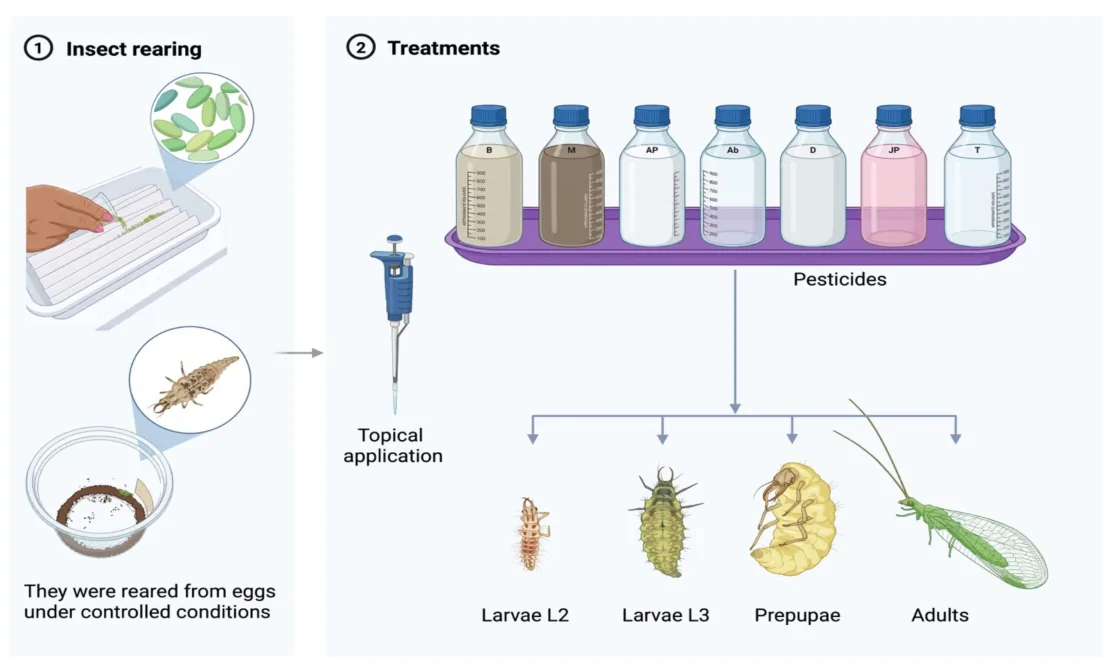
Methodological design to evaluate the lethal effects of biorational pesticides on *Chrysoperla carnea*. (**1**) Colony rearing: individuals were obtained from eggs laid on folded paper and fed with eggs of *Sitotroga cerealella* under controlled conditions. (**2**) Topical application on the second thoracic segment: six biorational pesticides (B, M, AP, Ab, JP, and D) and one control (water, T) were applied individually to four developmental stages: second-instar (L2) larvae, third-instar (L3) larvae, prepupae, and adults.

Four independent bioassays involving topical treatment application were conducted, each targeting a specific developmental stage of *C. carnea*: Bioassay A, second-instar (L2) larvae; Bioassay B, third-instar (L3) larvae; Bioassay C, prepupae; and Bioassay D, adults (Figure 1). Larvae were monitored daily to accurately identify each moult, and both L2 and L3 individuals were treated within 24 h after shedding their exuviae. For Bioassay C, treatments were applied during the prepupal stage, identified by the cessation of feeding and the characteristic C-shaped curvature of the body. For Bioassay D, adults were treated within 24 h of emergence, excluding individuals that still displayed pre-imaginal morphological characteristics. In all bioassays, only healthy individuals with similar appearances and body sizes were selected. A completely randomised design was used, with four replicates per treatment and developmental stage. Each experimental unit consisted of five individuals, resulting in a total of 20 individuals per treatment per stage.

From the first larval instar (L1), neonate larvae were individualised in acrylic pill boxes (4 cm × 1.3 cm) to prevent cannibalism and sterile *S. cerealella* eggs were offered ad libitum until the target stage was reached. Pill box lids were fitted with a small hole covered with organza fabric to provide ventilation.

Treatments were applied to recently moulted second- and third-instar larvae (<24 h old). Larvae were treated individually by topical application to the prothorax using a calibrated 0.5 μL micropipette (L2 and L3 larvae). For prepupae, the application was performed on the central dorsal surface using a calibrated 0.5 μL micropipette (BOECO model 9220100, Hamburgo, Germany). Adults were treated on the prothorax also using a calibrated 0.5 μL micropipette. Micropipette tips were changed between individuals to avoid cross-contamination. Twenty replicates per treatment were performed.

### 2.3. Sporulation Verification for Entomopathogenic Fungi Treatments

To verify fungal viability, *B. bassiana* and *M. anisopliae* were plated on Petri dishes containing potato dextrose agar (PDA) supplemented with 1% lactic acid and chloramphenicol to prevent bacterial contamination. Plates were incubated at 23 °C [28]. After 8 days, morphological identification was performed according to Barnett and Hunter [29].

To confirm sporulation, treated insect cadavers were surface-disinfected in 70% ethanol for 1 min and placed in moist chambers (Petri dishes with moist filter paper). The chambers were incubated at 27 °C for 10 days. Fungal mycelium was stained with methylene blue and aniline blue and examined by light microscopy at 10× and 40× magnifications.

### 2.4. Evaluation Variables

Effects on survival. Cumulative corrected mortality in L2 and L3 larvae was evaluated every 24 h until pupation. A larva was considered dead if it showed no movement. Pupal mortality was evaluated in an independent population; a pupa was considered dead if it turned dark. Adult mortality was evaluated for 8 consecutive days in an independent population, with data recorded every 24 h; individuals surviving this period were classified as survivors.

Effects on development. The period from treatment application to stage transition was evaluated for L2, L3 and prepupal bioassays. In adults, evaluation was performed for up to 8 days; exceeding 8 days was considered an indicator of survival.

### 2.5. Statistical Analysis

For each developmental stage and pesticide treatment, data were organised as raw counts of dead and surviving individuals per replicate. A generalised linear model (GLM) with a binomial distribution and logit link function was fitted to quantify the effect of pesticide treatment on mortality probability. Significance was assessed using likelihood ratio tests via an analysis of deviance (ANODEV, χ^2^ approximation). When significant, estimated marginal means (EMMs) and their standard errors (SEs) were obtained on the probability scale and pairwise comparisons were performed with Tukey’s adjustment, reporting compact letter displays. EMMs were expressed as corrected mortality percentage (±SE) and classified according to IOBC hazard categories: Class 1 = harmless (<30%); Class 2 = slightly harmful (30–79%); Class 3 = moderately harmful (80–99%); and Class 4 = harmful (>99%) [23,26].

Survival curves were estimated using the Kaplan–Meier method for each developmental stage and pesticide treatment. Differences in survival among treatments were assessed using the log-rank test, with pairwise comparisons adjusted using the false discovery rate (FDR) method.

The effect of pesticide treatment on developmental stage duration was evaluated using the Kruskal–Wallis test, followed by Dunn’s post hoc test with FDR *p*-value adjustment because assumptions of normality (Shapiro–Wilk test) and homogeneity of variances (Levene’s test) were not met. All statistical analyses were performed in R (version 4.5.1).

Three response variables were evaluated: corrected mortality, survival over time, and developmental stage duration. Corrected mortality was calculated using Abbott’s formula [30]:

$$Mc\;=\frac{Mt\;-\;Mc\;(control)}{100\;-\;Mc\;(control)}\times \;100$$

where *Mc* is the corrected mortality (%), *Mt* is the observed mortality (%) in the treatment, and *Mc* (*control*) is the observed mortality (%) in the control, to account for natural mortality in the control. Since the control (distilled water) served as the baseline used to remove its effect from the treatments, it was subsequently excluded from all inferential analyses.

### 2.6. Methodological Limitations

A key methodological consideration is that topical application, while standard for IOBC laboratory screening, does not replicate the nature or intensity of pesticide exposure that *C. carnea* would experience under field conditions. Direct application of 0.5 µL to the prothorax delivers a concentrated bolus dose to a localised body region, whereas field-exposed individuals typically encounter dried residues on plant surfaces, volatilised compounds, or diluted spray droplets, all of which result in substantially lower effective doses and different absorption kinetics [12,31]. Consequently, mortality rates obtained under topical exposure are expected to overestimate field-level risk, particularly for physical barrier products such as paraffinic oil and potassium soap, whose toxicity depends on surface contact phenomena that diminish rapidly as residues age and dry [32,33]. These results should therefore be interpreted as worst-case ecotoxicological benchmarks rather than direct predictors of population-level impacts in orchards. Field or semi-field validation studies, incorporating realistic exposure scenarios and population-level endpoints such as fecundity and prey consumption rates, are needed to confirm the practical compatibility of the evaluated products with *C. carnea* in avocado agroecosystems.

## 3. Results

### 3.1. Insect Mortality

**Figure 2:**
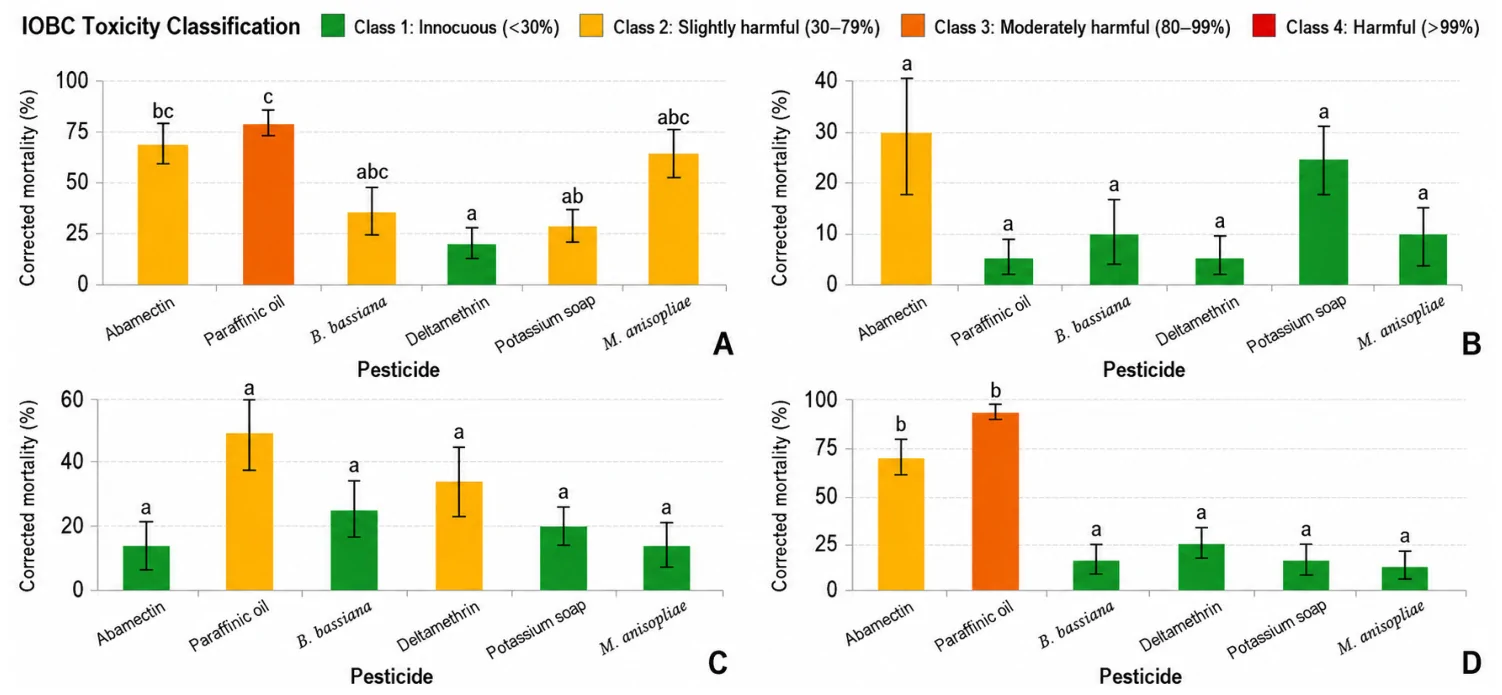
Mortality diagrams and IOBC toxicity categories for the different *Chrysoperla carnea* stages against six pesticide products. (**A**) L2 larvae, (**B**) L3 larvae, (**C**) pupae, and (**D**) adults. Means followed by different letters are significantly different at *p* < 0.05 according to Tukey’s test.

The effect of treatment was highly significant on the cumulative mortality of L2 larvae (*p* = 0.001; deviance = 25.65; Table 2; Figure 2A). Paraffinic oil induced the highest cumulative mortality (80.00%), followed by abamectin and *M. anisopliae*. On the other hand, *Beauveria bassiana* caused 35.00% cumulative mortality, potassium soap caused 30.00% and deltamethrin caused the lowest mortality. According to IOBC classification standards, paraffinic oil was moderately harmful (Class 3), abamectin and *M. anisopliae* were slightly harmful (Class 2), *B. bassiana* and potassium soap were slightly harmful (Class 2) and deltamethrin was harmless (Class 1) to L2 larvae.

In third-instar larvae, no statistically significant differences were found for the pesticide factor (*p* = 0.105; deviance = 9.09; Table 2; Figure 2B). Corrected cumulative mortality ranged from 5.00% (deltamethrin and paraffinic oil) to 30.00% (abamectin).

In the pupal stage, no significant differences were observed in corrected mortality (*p* = 0.10; deviance = 9.22; Table 2; Figure 2C). All treatments were harmless (Class 1), except for deltamethrin, which showed 35.00% mortality, corresponding to the slightly harmful category (Class 2). In adults, significant differences were observed (*p* = 0.001; deviance = 61.38; Table 2; Figure 2D). Paraffinic oil caused the highest mortality (91.60%), classified as moderately harmful (Class 3), followed by abamectin (70.80%), classified as slightly harmful (Class 2). The remaining treatments had mortality percentages below 30%, placing them in the harmless category (Class 1).

### 3.2. Temporal Dynamics of Mortality

**Figure 3:**
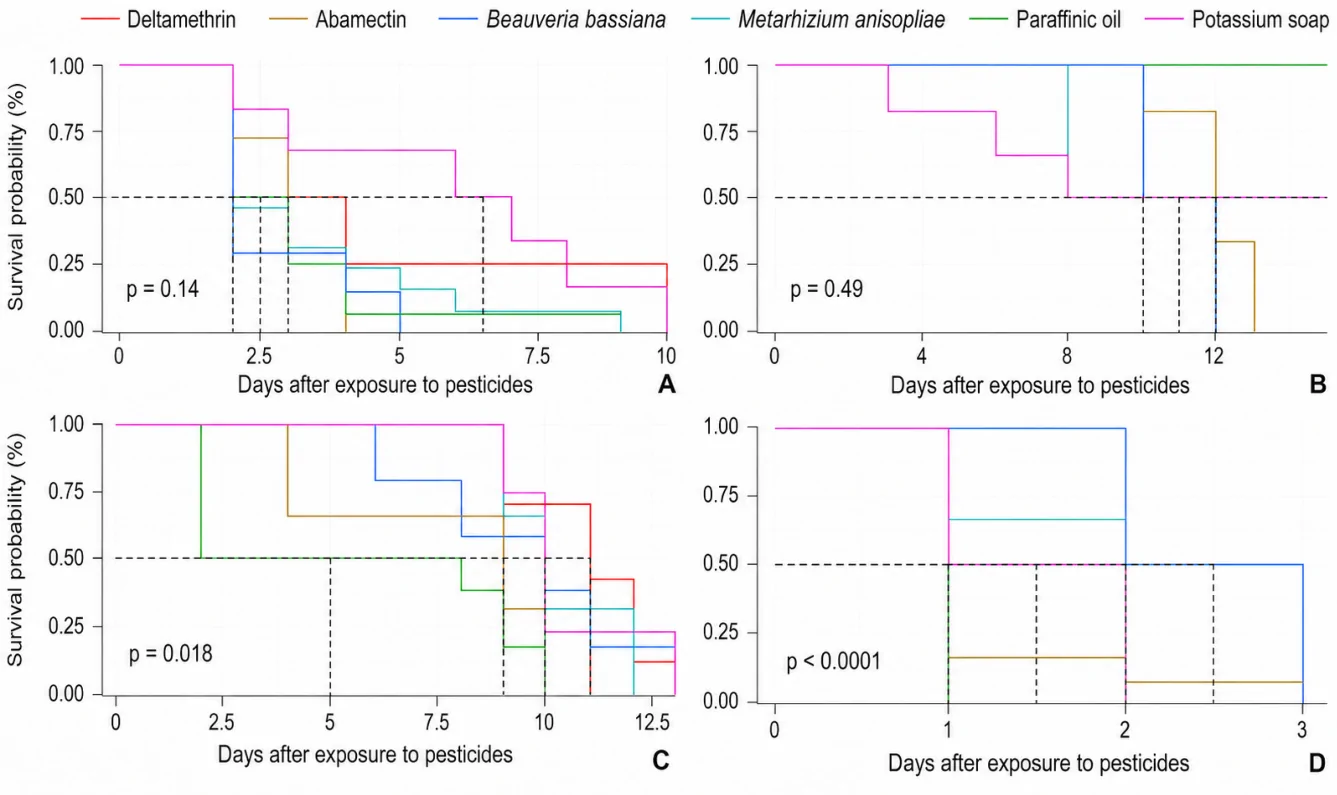
Kaplan–Meier curves of the temporal dynamics of *Chrysoperla carnea* mortality when exposed to six pesticide products used in avocado cultivation. (**A**) L2 larvae, (**B**) L3 larvae, (**C**) pupae, and (**D**) adults.

In L2 larvae, Kaplan–Meier curves revealed biologically relevant differences in survival patterns despite a non-significant global log-rank test (*p* = 0.14; Figure 3A). Entomopathogenic fungi (*B. bassiana* and *M. anisopliae*) and paraffinic oil produced markedly reduced survival during the first five days, suggesting acute and sustained toxic effects. Abamectin also showed early decline, albeit with a less abrupt pattern. Potassium soap showed a weaker impact with more gradual survival decreases.

In L3 larvae, most pesticides exerted comparatively moderate effects on survival (*p* = 0.49; Figure 3B). Larvae exposed to paraffinic oil and *B. bassiana* maintained survival near 100% throughout most of the bioassay, suggesting low acute toxicity at this stage. Larvae exposed to abamectin showed a progressive decline after day 10 and *M. anisopliae* caused an abrupt drop around day 8. Potassium soap caused an early reduction around day 5 but the survival rate subsequently stabilised at near 50% by the end of the bioassay.

For the pupal stage, more pronounced differences were observed, which were statistically significant according to the log-rank test (*p* = 0.018; Figure 3C). Paraffinic oil caused an abrupt decline around day 2, rapidly reaching 50% survival. Entomopathogenic fungi resulted in progressive mortality throughout the bioassay, reaching approximately 20–30% survival by day 12. Larvae exposed to deltamethrin maintained a relatively high survival rate until day 9 before it declined sharply, while abamectin caused a moderate and consistent decline in mortality. Potassium soap was the most stable treatment, with larvae maintaining a survival rate above 75% until day 9.

In adult *C. carnea*, the strongest treatment effects were observed (*p* < 0.0001; Figure 3D), with survival declining sharply within the first two days of exposure for most treatments. Only the adults exposed to potassium soap maintained a survival rate above 50% beyond day 2.

The results showed significant differences in L2 larval duration (*p* = 0.0001; H = 30.38) and adult longevity (*p* = 0.001; H = 21.25), while no differences were detected in L3 (*p* = 0.53) or pupal duration (*p* = 0.28; Table 3). In L2 larvae, the control represented the optimal development time for this instar (2.83 ± 0.19 days), whereas all pesticide treatments significantly prolonged the duration of this stage (4.33–4.82 days). In adults, longevity differed significantly among treatments; deltamethrin caused the greatest reduction in longevity (6.25 ± 0.75 days), whereas adults under all other treatments showed a mean longevity of 8.0 ± 0.0 days, comparable to the control.

## 4. Discussion

Paraffinic oil was classified as moderately harmful to L2 larvae, indicating an important risk for the survival of natural enemies such as *C. carnea*. Many mineral or paraffinic oils, although considered biorational products, may obstruct cuticular respiration, alter the wax layer and cause asphyxiation or dehydration in small insects [33]. Unlike synthetic insecticides, petroleum oil does not appear to bind to specific receptors [34] and its toxicity depends on surface phenomena, biochemical traits, taxa and the developmental stage of the target insect [32].

Abamectin produced a 70% mortality rate in L2 larvae, contrasting with the results of Mohammed and colleagues [35], who reported 34 ± 7.48% mortality using a residual contact method rather than topical application. The more intense direct contact in the present study likely explains the different mortality responses.

*Metarhizium anisopliae*, at 1 × 10^9^ CFU/mL, produced a 65% corrected cumulative mortality rate in L2 larvae, a level that would be considered positive for the biocontrol of pest insects but warrants caution in the context of natural enemy conservation [36]. This concern is further supported by Thungrabeab and Tongma [37], who evaluated the non-target effects of *B. bassiana* and *M. anisopliae* on natural enemies including *Coccinella septempunctata* Linnaeus (Coleoptera: Coccinellidae), *Chrysoperla carnea*, and *Dicyphus tamaninii* Wagner (Hemiptera: Miridae) at a concentration of 1 × 10^8^ conidia/mL. Their results showed that *B. bassiana* was non-pathogenic to these natural enemies and to the beneficial soil-dwelling springtail *Heteromurus nitidus* Templeton (Entomobryomorpha: Entomobryidae), whereas *M. anisopliae* exhibited pathogenicity toward the natural enemies tested, including *C. carnea*, at the first larval instar stage.

*Beauveria bassiana* resulted in 35% corrected cumulative mortality in L2 larvae, and was thus classified as slightly harmful, similar to the results of Imam [38], who reported 39.17% corrected mortality at a lower concentration (1 × 10^7^ CFU/mL) applied residually. The unusually low mortality observed with deltamethrin (20%) contrasts with studies describing pyrethroids as highly toxic to chrysopids [11,12] and may reflect physiological tolerance in the evaluated population as chrysopid larvae possess high levels of pyrethroid esterase activity [39].

The results for the L3 larvae are similar to the documented pattern of increasing tolerance with larval age. Mingotti and colleagues [19] showed that *B. bassiana* induced significant mortality only in first-instar *C. externa* larvae (26% at 120 h), with progressively weaker effects on second (17%) and third instars (10%). The strain-specific nature of fungal virulence is also relevant, with different *B. bassiana* strains showing variable susceptibility among chrysopid species and developmental stages. This age-dependent tolerance may be partly associated with developmental changes in cuticular architecture and composition. Before reaching the hemocoel, entomopathogenic fungi such as *B. bassiana* must adhere to and penetrate the cuticle, a non-cellular extracellular matrix secreted by the underlying epidermis. The procuticle consists of chitin microfibrils embedded in a protein-rich matrix, whereas the epicuticle contains proteins, lipids, and other compounds but lacks chitin [40]. During cuticle maturation, tanning and sclerotisation involve the quinone-mediated cross-linking of cuticular proteins, increasing cuticular rigidity. Therefore, developmental differences in cuticle thickness, composition, and degree of sclerotisation could contribute to the lower susceptibility of later instars to fungal penetration, although these characteristics were not directly measured in the present study [41].

In adults, paraffinic oil and abamectin caused the highest mortality. The rapid toxic action of paraffinic oils on adults is consistent with their physical mode of action—penetration of cuticular waxes and disruption of water regulation [32]. Regarding potassium soap, Worth and colleagues reported 15% corrected mortality, consistent with our result of 16.00% [42]. Portilla and colleagues reported that *B. bassiana* has lethal effects on *Chrysoperla rufilabris* Burmeister (Neuroptera: Chrysopidae) with the severity increasing in a dose-dependent manner [43].

The prolongation of L2 larval duration observed in all treatments relative to the control indicates a systemic sublethal effect on *C. carnea* larval physiology. This response has been widely described in beneficial insects exposed to neurotoxic insecticides or growth regulators, where sublethal exposure alters hormonal balance (ecdysone and juvenile hormone) or interferes with chitin synthesis, affecting the moulting process [44,45]. The result is delayed development without immediate mortality, potentially compromising predator–prey synchronisation.

In adults, the reduction in longevity induced by deltamethrin (6.25 ± 0.75 days vs. 8.0 days in the control) suggests delayed physiological effects that are expressed in adulthood. Pyrethroids act on voltage-gated sodium channels, prolonging neuronal depolarisation and causing hyperexcitation, oxidative stress and mitochondrial dysfunction, potentially reducing life expectancy even in surviving individuals [46]. Larval exposure may also alter detoxifying enzyme activity, affecting adult energy homeostasis [47]. These results agree with those of Garzón and colleagues [12], who reported that pyrethroid exposure reduced adult longevity and fecundity in *C. carnea* even in the absence of high larval mortality.

The compatibility between biopesticides and natural enemies depends critically on the predator species, developmental stage, fungal strain, dose, formulation and exposure route [48]. The target pest’s ecology and the characteristics of the production system also influence management decisions. In ornamental crops, where aesthetic damage and quarantine restrictions result in very low pest tolerance, early or preventive interventions may be particularly important [49]. Studies on *Chrysoperla lucasina* (Lacroix), a close relative of *C. carnea*, found that *B. bassiana* caused only slight differences in survival and developmental time relative to untreated controls [50]. These findings contrast with the more pronounced effects observed in our study, suggesting that direct topical exposure amplifies toxicity relative to residual or indirect contact methods. Similarly, Ríos-Moreno and colleagues [51] confirmed that *Metarhizium brunneum* Petch strains had a low toxicity risk for *C. carnea* under greenhouse conditions, though secondary metabolites such as destruxin A were detected at low concentrations in predator tissues, raising questions about long-term sublethal effects.

Overall, the survival patterns observed across developmental stages reinforce that risk assessments of biorational pesticides on chrysopids must be conducted stage-by-stage, as toxicity cannot be inferred from results obtained in a single developmental stage [52,53]. The present study provides evidence that exposure timing relative to *C. carnea* life stages is a critical factor in designing IPM programmes for avocado that incorporate this predator.

## 5. Conclusions

This study demonstrates that the ecotoxicological risk posed by synthetic insecticides, entomopathogenic fungi, and physical barrier products to *C. carnea* cannot be characterised based on a single developmental stage or a single toxicity endpoint. By evaluating four life stages under a standardised topical bioassay, this work confirms that early larval instars represent the critical exposure window for this predator, whereas later stages display comparatively higher tolerance; consequently, pesticide compatibility ratings derived from adult or single-stage assays risk underestimating the true impact on population-level biological control.

Second-instar larvae emerged as the most vulnerable life stage, exhibiting the highest cumulative mortality under paraffinic oil and abamectin exposure, alongside a consistent prolongation of developmental duration across all treatments relative to the control. This pattern indicates that early larval instars represent a critical window of ecological vulnerability, during which even sublethal pesticide exposure may compromise the predator’s developmental trajectory and, by extension, its capacity to provide effective biological control. In contrast, third-instar larvae and pupae displayed markedly greater tolerance, with most products falling within the harmless IOBC category, a finding consistent with the broader entomological literature describing an age-related increase in xenobiotic tolerance among chrysopid larvae, likely mediated by enhanced detoxification enzyme activity and cuticular thickening.

The finding that *M. anisopliae* was less selective toward *C. carnea* than *B. bassiana* challenges the general assumption that entomopathogenic fungi are inherently compatible with natural enemies in IPM programmes, and underscores that fungal biopesticides must be evaluated and selected at the strain level rather than assumed safe based on their microbial origin. Likewise, the sublethal prolongation of larval development observed across nearly all treatments, independent of acute mortality, indicates that IOBC lethality classifications alone are insufficient to capture the full ecological cost of exposing *C. carnea* to pesticides; disruption of developmental timing can desynchronise predator–prey dynamics even when direct mortality is low.

These results support the design of stage- and product-specific compatibility protocols, rather than blanket recommendations, for integrating *C. carnea* into IPM strategies for avocado. Practical implications include prioritising the use of potassium soap and B. bassiana during early larval stages when feasible, restricting paraffinic oil and abamectin applications to periods when *C. carnea* larvae are absent or in later, more tolerant instars, and screening candidate *M. anisopliae* strains for non-target selectivity before field deployment.

Future research should extend these laboratory findings to greenhouse and field conditions, incorporate additional sublethal endpoints such as fecundity and predation efficiency, and evaluate strain-level variability in entomopathogenic fungi to refine risk assessment frameworks for chrysopid conservation in Neotropical avocado agroecosystems.

## Figures and Tables

**Table 1:** Identity, dosage, and mode of action of biorational pesticides and the control treatment evaluated in four bioassays on *Chrysoperla carnea*.

Product	Dosage *	Trade Name	Formulation	Manufacturer	Mode of Action	Concentration/CFU
Deltamethrin	0.3 L/ha	Decis^®^ Forte	Emulsifiable concentrate	Bayer^®^ (Veracruz, Mexico)	Neurotoxic (sodium channels)	100 g/L
Abamectin	1.2 L/ha	Agrimec^®^	Emulsifiable concentrate	Syngenta^®^ (San Luis, Potosí, Mexico)	Neurotoxic (GABA)	1.8% (18 g/L)
Paraffinic oil	2 L/ha	SAF-T-SIDE^®^	Pre-emulsified oil	BRANDT^®^ (CDMX, Mexico)	Spiracle blockage	80% (800 g/L)
Potassium soap	2 L/ha	Agronortech^®^	Liquid	Agronortech^®^ (Texcoco, Mexico)	Membrane disruption	35 g/L
*Beauveria bassiana*	480 g/ha	BEATRON^®^	Wettable powder	Plant Health Care (Querétaro, Mexico)	Cuticular penetration and toxins	≥1 × 10^9^ CFU/g
*Metarhizium* *anisopliae*	480 g/ha	METATRON^®^	Wettable powder	Plant Health Care (Querétaro, Mexico)	Cuticular penetration and toxins	≥1 × 10^9^ CFU/g
Control (water)	N/A	—	Distilled water	—	—	—

* Maximum label-recommended field rate. Doses per hectare assume an application volume of 200 L of water.

**Table 2:** Accumulated mortality (%) in the different developmental stages of *Chrysoperla carnea* against six pesticide products.

Applied Product	Development Stages of *C. carnea*			
	L2 Larvae	L3 Larvae	Pupae	Adult
	Mortality (%) SE	Mortality (%) SE	Mortality (%) SE	Mortality (%) SE
Deltamethrin	20.00 ± 0.08 a	5.00 ± 0.04 a	35.00 ± 0.10 a	25.00 ± 0.08 a
Abamectin	70.00 ± 0.10 bc	30.00 ± 0.10 a	15.00 ± 0.07 a	70.80 ± 0.09 b
*B. bassiana*	35.00 ± 0.10 abc	10.00 ± 0.06 a	25.00 ± 0.09 a	16.00 ± 0.07 a
*M. anisopliae*	65.00 ± 0.11 abc	10.00 ± 0.06 a	15.00 ± 0.07 a	12.50 ± 0.06 a
Potassium soap	30.00 ± 0.10 ab	25.00 ± 0.09 a	20.00 ± 0.08 a	16.00 ± 0.07 a
Paraffinic oil	80.00 ± 0.08 c	5.00 ± 0.04 a	50.00 ± 0.11 a	91.60 ± 0.05 b
N	20	20	20	24
*p* value	0.001 **	0.105 ns	0.10 ns	0.001 **
Deviance	25.65	9.09	9.22	61.38

Means followed by different letters are significantly different at *p* < 0.05 according to Tukey’s test. SE: standard error. ** statistical difference. ns: not significance. IOBC hazard categories: Class 1 = harmless (<30% mortality); Class 2 = slightly harmful (30–79% mortality); Class 3 = moderately harmful (80–99% mortality); and Class 4 = harmful (>99% mortality).

**Table 3:** Development time of different *Chrysoperla carnea* stages when exposed to six pesticide products used in avocado cultivation. Different letters indicate significant differences between treatments for each developmental stage.

Treatment	Development Stages of *C. carnea*			
	L2 Larvae	L3 Larvae	Pupae	Adults
	(Days)	(Days)	(Days)	(Days)
Abamectin	4.33 ± 0.42 a	4.63 ± 0.38 a	7.23 ± 0.25 a	8.0 ± 0.0 a
*Beauveria bassiana*	4.62 ± 0.26 a	4.76 ± 0.40 a	7.03 ± 0.23 a	8.0 ± 0.0 a
Control	2.83 ± 0.19 b	4.89 ± 0.40 a	7.15 ± 0.20 a	8.0 ± 0.0 a
Deltamethrin	4.82 ± 0.28 a	4.92 ± 0.36 a	6.87 ± 0.28 a	6.25 ± 0.75 b
*Metarhizium anisopliae*	4.67 ± 0.21 a	4.89 ± 0.35 a	7.53 ± 0.26 a	8.0 ± 0.0 a
Paraffinic oil	4.50 ± 0.28 a	5.41 ± 0.42 a	7.58 ± 0.21 a	8.0 ± 0.0 a
Potassium soap	4.43 ± 0.22 a	4.53 ± 0.35 a	7.11 ± 0.23 a	8.0 ± 0.0 a
Kruskal–Wallis Chi-squared	30.38	5.03	7.41	21.25
*p* value	0.0001 **	ns	ns	0.001 **

Means followed by different letters are significantly different at *p* < 0.05 according to Dunn’s test. SE: standard error. ** statistical difference; ns: not significance.

## Data Availability

Data will be made available on request. During the preparation of this work, Artificial intelligence tools were used in the preparation of this manuscript. Specifically, Claude Sonnet 4.6 (Anthropic, San Francisco, CA, USA) was employed for language editing of the document, while all scientific content, data interpretation, and conclusions remained solely the responsibility of the authors. Figures presented in this study were created using BioRender (BioRender.com), an AI-powered scientific illustration platform, based on original photographs taken by the authors during the experimental procedures.

## Abbreviations

The following abbreviations are used in this manuscript:

IPM	Integrated pest management
FDR	False discovery rate
SE	Standard error
IOBC	International Organisation for Biological Control
